# CSF oligoclonal IgG bands are not associated with ALS progression and prognosis

**DOI:** 10.3389/fneur.2023.1170360

**Published:** 2023-05-05

**Authors:** Veronika Klose, Sarah Jesse, Jan Lewerenz, Jan Kassubek, Johannes Dorst, Hayrettin Tumani, Albert C. Ludolph, Francesco Roselli

**Affiliations:** ^1^Department of Neurology, Ulm University, Ulm, Germany; ^2^German Center for Neurodegenerative Diseases (DZNE)-Ulm, Ulm, Germany; ^3^Neurozentrum Ulm, Ulm, Germany

**Keywords:** oligoclonal bands, intrathecal synthesis, age of onset, survival, cerebrospinal fluid, amyotrophic lateral sclerosis

## Abstract

**Introduction:**

Amyotrophic Lateral Sclerosis (ALS) is characterized by progressive motoneuron degeneration through cell autonomous and non-cell autonomous mechanisms; and the involvement of the innate and adaptive immune system has been hypothesized based on human and murine model data. We have explored if B-cell activation and IgG responses, as detected by IgG Oligoclonal bands (OCB) in serum and cerebrospinal fluid, were associated with ALS or with a subgroup of patients with distinct clinical features.

**Methods:**

IgG OCB were determined in patients affected by ALS (n=457), Alzheimer Disease (n=516), Mild Cognitive Impairment (n=91), Tension-type Headache (n=152) and idiopathic Facial Palsy (n=94). For ALS patients, clinico-demographic and survival data were prospectively collected in the Register Schabia.

**Results:**

The prevalence of IgG OCB is comparable in ALS and the four neurological cohorts. When the OCB pattern was considered (highlighting either intrathecal or systemic B-cells activation), no effect of OCB pattern on clinic-demographic parameters and overall. ALS patients with intrathecal IgG synthesis (type 2 and 3) were more likely to display infectious, inflammatory or systemic autoimmune conditions.

**Discussion:**

These data suggest that OCB are not related to ALS pathophysiology but rather are a finding possibly indicative a coincidental infectious or inflammatory comorbidity that merits further investigation.

## Introduction

Amyotrophic lateral sclerosis (ALS) has been originally conceptualized to be primarily a motoneuron disease, based on clinical, pathological and experimental evidence (1). However, recent evidence points toward a multidimensional pathophysiology (2) which may involve systemic (3–5) or CNS-restricted (6–8) immune responses. Alterations have been described in several cellular immunity compartment: disturbances in CD4+ lymphocytes (9) and Th17 (10)correlate with disease progression (11). Recently, also disturbances in B-cell dependent, humoral immunity have been suggested: meningeal tertiary lymphoid-like structures containing B-cells have been identified in a ALS murine model (12). Autoantibodies against multiple neuronal antigens (13–15) have been reported in ALS patients, and antibody-mediated toxicity in ALS has been proposed based on IgG transfer to animals (16, 17); the pathophysiological relevance of these observations remains to be ascertained. To date, the question of an ongoing B-cell response in ALS patients, or in a subset of them, remains open. Given the availability of anti-B-cell treatments [e.g., anti CD19 and CD20 monoclonal antibodies; (18)] and the initial testing of rituximab in ALS trials, addressing this issue may shape the clinical trial landscape. More broadly, no immune response marker has gained widespread use in clinical practice for prognostication or patient stratification (19).

The detection of IgG-specific oligoclonal bands (OCB) in cerebro-spinal fluid (CSF) has been extensively employed to identify an active immune response in the form of the expansion of a limited number of B-cell clones (20) associated with extrathecal or intrathecal IgG synthesis (21). OCB is commonly associated with the diagnosis and pathophysiology of Multiple Sclerosis (22); however, OCB detection is reported also in infective, inflammatory and autoimmune conditions such as systemic lupus erythematosus, neurosyphilis, Behcet’s disease, neuroborreliosis, aseptic meningitis, Sjögren’s syndrome, autoimmune encephalitis and CNS vasculitis (23), and therefore OCBs are a bona-fide marker of immune activation irrespective of the etiology.

The comparison of OCB in serum and CSF allows the distinction of 5 patterns (24, 25): absence of OCB in both CSF and serum (type 1, no immune activation), presence of OCB in CSF but not in serum (type 2, indicating CNS-restricted B-cells activation), presence of some OCBs in CSF and in serum, with in addition OCB detected in CSF but not in serum (type 3, indicating a systemic B-cell activation together with a component restricted to the CNS), presence of the same OCBs in serum and CSF (type 4, pointing toward systemic B-cell activation without specific CNS components) and strong monoclonal OCB detected both in CSF and serum (type 5, usually related to monoclonal gammopathies). Thus, OCB provides a window to the ongoing extrathecal (systemic) as well as intrathecal (CNS-restricted) humoral immune activation and immunoglobulin synthesis.

Several initial studies (26, 27) suggested the possibility of increased prevalence of OCB in ALS patients, although diagnostic uncertainties at the time left the issue unresolved. More recent work (28) suggests that OCB may identify a subset of ALS patients with distinct genetic or clinical characteristics; however, a large-scale investigation of this hypothesis has not yet been performed.

The aim of our study was to address (a) whether there is any B-cell activation in ALS patients, or in a subset of them, related to the immune system activation and relevant to the pathogenic process; and (b) whether OCB patterns may identify a subset of ALS patients with distinct clinical and prognostic characteristics [as previously suggested for different immune markers-(29)]. We set out to address these questions by investigating the CSF data from a large (>450 patients) cohort of ALS patients prospectively recruited and followed-up at Ulm University.

## Methods

### Patients

The present study was performed in agreement to the Helsinki protocol on human experimentation and was approved by Ethics committee of the Ulm University, No. 20/10.

In the present study, five cohorts of patients, prospectively recruited at the Department of Neurology of Ulm University (Ulm, Germany) were taken into consideration.

Amyotrophic lateral sclerosis patients were diagnosed according to the revised El Escorial criteria (30). Inclusion criteria included (i) diagnosis of suspected, possible, probable and definite ALS reported by the ALS Registry Swabia (ii) age of onset >16 years (to limit the erroneous inclusion of SMA patients due to mis-reporting in the clinical charts) (iii) Exclusion criteria (i) final diagnosis of SMA or HSP (ii) age of onset <15.

The progression rate is calculated either *(ALS-FRS atfirstvisit—ALS-FRS at last visit)/months between those measurements* (250 patients) OR if only one visit was documented *(48—ALS-FRS at first visit)/months between onset and visit*, as previously reported (31–33).

Patients with tension-type headache (TH) were diagnosed according to International Classification of Headache Disorders-3rd edition (ICHD-3; https://ichd-3.org/) after clinical assessment, neuroimaging evaluation and blood and CSF parameters consideration.

Patients with Alzheimer disease (AD) and Mild cognitive impairment (MCI) were diagnosed according to current criteria (34, 35). In addition to anamnesis and third-party anamnesis, clinical neurological examination and laboratory for exclusion diagnostics, we included also neuropsychological testing and CSF determination of dementia markers total tau, pTau181, Ab42, Aβ 40/42 ratio in CSF were measured using Fujirebio, Gent, Belgium.

Patients with peripheral idiopathic facial palsy (FP) was diagnosed according to the German guidelines for the diagnosis of idiopathic facial nerve palsy (36, 37). For each patient, secondary causes of FP (37) were excluded by a combination of clinical examination and MRI; therefore, non-idiopathic FP cases (38) were excluded.

### CSF sampling and processing

Cerebro-spinal fluid was obtained and analyzed as previously reported (39, 40). Upon lumbar puncture, CSF samples were collected in polypropylene tubes and subject to analysis within 1 h from sampling. Leukocyte count was performed by trained operators employing phase-contrast microscopy and a Fuchs–Rosenthal chamber as previously reported (40). CSF samples with contamination of erythrocytes were excluded in order to avoid false positive values of CSF albumin and lactate. Total protein, albumin, IgG, IgA, and IgM were measured by standard immunochemical nephelometry in CSF and serum [Dade–Behring nephelometer analyzer (ProSpec), Marburg, Germany] using a polyclonal antibody in the case of albumin and IgG as well as a latex particle-amplified antibody reaction in the cases of IgA. The inter- and intra-assay variabilities were <10% for the method-dependent absolute levels as well as for the method-independent CSF/serum quotients of albumin, IgG, IgA. CSF lactate was determined by a lactate–oxidase reaction (Greiner GmbH, Flacht, Germany).

### Oligoclonal bands detection

Oligoclonal bands were determined by isoelectric focusing (IEF) on polyacrylamide gels followed by immunoblotting using an IgG-specific antibody staining, as previously reported (41). Briefly, CSF and serum samples from the same patient were adjusted for IgG concentrations and run on the same gel. Two long-standing experienced technicians and at two board-certified neurologists with extensive experience in the field of CSF analysis independently scored the immunoblots; consensus over the subtype was reached before the subtype was assigned to the dataset. The classification of the OCB subtype was performed as previously reported (21, 24, 25): type 1-no oligoclonal IgG band, normal finding; type 2-Isolated OCB (2 or more IgG bands) in the CSF; type 3-Identical OCB (2 or more) in cerebrospinal fluid and serum, additionally (1 or more) isolated OCB in the CSF; Type-4: OCB with identical (mirror image) distribution in the CSF and serum; type 5-Monoclonal bands with identical distribution in the CSF and serum (usually suggesting a systemic gammopathy). Representative images of the five patterns as seen in ALS patients are provided in Supplementary Figure 1.

### Statistical analysis

**Figure 1:**
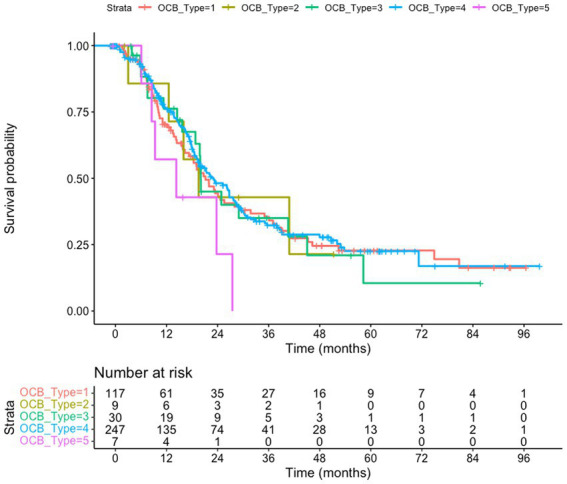
Survival of amyotrophic lateral sclerosis (ALS) patients subgrouped according to the oligoclonal bands (OCB) subtype. The whole cohort (*n* = 457) is taken into consideration, using the date of the last visit as endpoint. No difference in survival is observed (*p* = 0.46); patients with type 5 pattern have a trend toward shorter survival compared to type 4 (*p* = 0.064). Kaplan–Meyer log-rank test.

**Figure 2:**
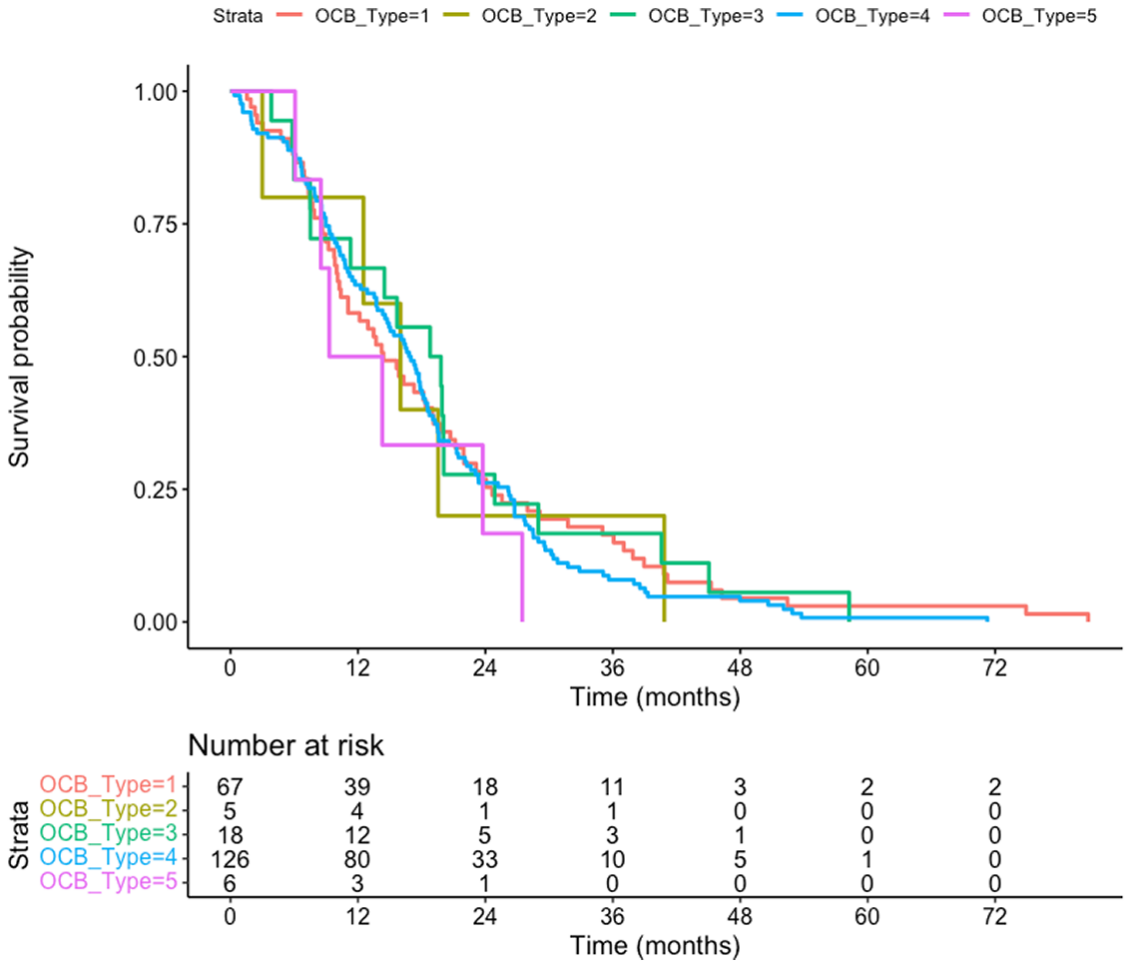
Death-certificate survival of amyotrophic lateral sclerosis (ALS) patients subgrouped according to the oligoclonal bands (OCB) subtype. The subgroup of ALS patients for whom death certificates are available (*n* = 222) is considered; no difference of survival according to the OCB subtype is observed (*p* = 0.83). Kaplan–Meyer log-rank test

The statistical analyses were performed using the R software (version 4.0.3) on RStudio (version 1.2.5033). For the comparison of clinic-demographic and CSF parameters across the five disease cohorts (ALS, AD, MCI, TTH, and FP), the Pearson’s chi-square test was used for categorical variables and one-way Analysis of Variance (ANOVA) with *post hoc* Tukey HSD (honestly significant difference) test was used for continuous variables (Table 1). Prevalence of OCB was analyzed by Pearson’s chi-square (Table 2). The comparison of clinico-demographic and CSF features (Tables 3, 4) across ALS subgroups with distinct OCB subtypes was performed with the Pearson’s chi-square test for categorical variables and one-way ANOVA with *post hoc* Tukey HSD (honestly significant difference) test for continuous variables. The prevalence of comorbidities (Table 5) was analyzed by Pearson’s chi-square test. The difference in survival of the five subgroups of ALS patients according to the OCB subtype was analyzed by Kaplan–Meyer log-rank test (Figures 1, 2); multivariate Cox regression analysis was used for assessing the role of possible confounders. In all the analyses we considered *p* = 0.05 as the level of significance after correction for multiple comparisons.

**Table 1:** Clinico-demographic and cerebro-spinal fluid (CSF) parameters of the Amyotrophic lateral sclerosis (ALS) population and of four neurological control groups (AD, MCI, TTH, and FP).

	ALS	AD	MCI	TTH	FP	*p*-Value
Number	457	516	91	152	94	
Females/males	185/272 (40%/60%)	319/197 (62%/38%)	51/40 (56%/44%)	98/54 (64%/36%)	60/34 (64%/36%)	*1.337e-11
Mean age (years) at sampling (SD)	62.29 (12.08)	69.38 (11.19)	68.58 (9.68)	48.05 (18.41)	52.39 (18.69)	*<2.200e-16
CSF Alb (mg/L) (SD)	292.27 (117.72)	298.74 (158.48)	287.69 (97.31)	251.56 (103.17)	276.12 (130.18)	**0.0041
Q-Alb × 10^3^ (SD)	6.94 (2.66)	7.54 (4.71)	6.66 (2.27)	5.89 (2.32)	6.49 (3.19)	**1.041e-05
CSF IgG (mg/L) (SD)	32.13 (14.49)	33.92 (18.53)	31.89 (14.87)	30.77 (17.24)	30.73 (15.35)	**0.1533
Q-IgG × 10^3^ (SD)	3.25 (1.34)	3.51 (2.07)	3.06 (1.03)	2.91 (1.67)	3.06 (1.53)	**0.0007
CSF IgA (mg/L) (SD)	4.25 (2.91)	4.91 (4.34)	3.92 (2.80)	4.10 (3.95)	3.85 (2.91)	**0.0046
Q-IgA × 10^3^ (SD)	1.90 (2.29)	1.90 (1.31)	1.60 (0.70)	1.64 (1.52)	1.69 (1.14)	**0.2349
CSF IgM (mg/L) (SD)	0.35 (0.39)	0.38 (0.70)	0.26 (0.21)	0.32 (0.40)	0.33 (0.65)	**0.3578
Q-IgM × 10^3^ (SD)	0.33 (0.23)	0.40 (0.51)	0.30 (0.17)	0.34 (0.70)	0.35 (0.37)	**0.0669
Leukocytes/μl (SD)	0.86 (1.23)	4.86 (43.23)	0.88 (1.00)	1.26 (1.48)	1.27 (1.17)	**0.1759
Number of Patients Leukocytes >5/μl (%)	4 (1%)	18 (4%)	0	4 (3%)	0	** 0.2434
Lactate (mmol/L) (SD)	1.79 (0.27)	1.84 (0.38)	1.72 (0.22)	1.77 (0.42)	1.82 (0.46)	**0.0076

The TTH and FP groups are significantly younger than the AD, MCI, and ALS. TTH patients have a significantly lower CSF Albumin and Q-Alb and Q-IgG; AD patients have a significantly higher Q-IgA. Lactate levels are significantly lower in ALS, MCI and TTH. *Pearson’s *χ*^2^ test; **One-way ANOVA, Tukey *post hoc* test.

**Table 2:** Comparable prevalence of oligoclonal bands (OCB) patterns in amyotrophic lateral sclerosis (ALS) and four neurological control populations.

	ALS	AD	MCI	TTH	FP	*p*-Value
OCB type 1 (%)	130 (28%)	106 (21%)	18 (20%)	42 (28%)	26 (28%)	*0.2237
OCB type 2 (%)	9 (2%)	8 (2%)	0	1 (1%)	0	*0.2458
OCB type 3 (%)	35 (8%)	47 (9%)	7 (8%)	13 (9%)	0	*0.2205
OCB type 4 (%)	275 (60%)	348 (67%)	65 (71%)	90 (59%)	66 (70%)	*0.2278
OCB type 5 (%)	8 (2%)	7 (1%)	1 (1%)	6 (4%)	2 (2%)	*0.2232

No difference in the prevalence of the different OCB types was detected across the five cohorts of patients. *Pearson’s *χ*^2^ test.

**Table 3:** Clinico-demographic features of amyotrophic lateral sclerosis (ALS) patients subgrouped according to the oligoclonal bands (OCB) pattern.

ALS patients (*n* = 457)							
		OCB type 1	OCB type 2	OCB type 3	OCB type 4	OCB type 5	*p*-Value
Number		130 (28%)	9 (2%)	35 (8%)	275(60%)	8 (2%)	*<2.2e-16
Female/male		56/74	3/6	16/19	106/169	4/4	*0.7942
Age of onset		58.38 (11.54)	57.93 (9.92)	62.43 (14.02)	61.29 (12.65)	67.07 (7.03)	**0.0776
Age at sampling		59.94 (11.40)	59.33 (9.93)	63.65 (14.06)	63.11 (12.15)	69.63 (7.44)	**0.0378
Site of onset:	Bulbar	31	4	13	71	3	*0.2380
	Spinal	94	4	21	191	5	
	Uncertain	4	1	0	4	0	
BMI		24.81 (4.17)	25.01 (5.98)	25.86 (5.93)	24.66 (3.90)	25.60 (6.42)	**0.6301
ALS-FRS at sampling		40.7 (6.08)	42.56 (4.53)	36.97 (6.51)	40.21 (6.64)	38.63 (4.63)	**0.0369
Progression rate		0.94 (1.22)	1.08 (0.75)	1.09 (0.86)	0.81 (0.97)	1.20 (1.77)	**0.4332

Age at sampling was significantly different across groups with patients with type 5 OCB being significantly older than the others; ALS-FRS was significantly different across the groups, with *post hoc* test revealing a significant difference between OCB type 3 patients and OCB type 1 (*p* = 0.03) and OCB type 4 (with *p* = 0.06). *Pearson’s *χ*^2^ test; **One-way ANOVA, Tukey *post hoc* test.

**Table 4:** Cerebro-spinal fluid (CSF) parameters of amyotrophic lateral sclerosis (ALS) patients subgrouped according to the oligoclonal bands (OCB) pattern.

ALS patients (*n* = 457)						
	OCB type 1	OCB type 2	OCB type 3	OCB type 4	OCB type 5	*p*-Value
CSF Alb (mg/L)	299.15 (118.99)	320.44 (140.75)	274.29 (108.09)	292.30 (118.46)	225.50 (69.76)	0.3666
Q-Alb × 10^3^	6.95 (2.58)	7.32 (3.32)	6.79 (2.78)	6.98 (2.69)	5.50 (1.33)	0.6118
CSF IgG (mg/L)	32.20 (14.21)	30.60 (13.27)	34.06 (13.98)	32.05 (14.83)	27.34 (12.39)	0.8120
Q-IgG × 10^3^	3.24 (1.39)	3.63 (1.72)	3.43 (1.29)	3.24 (1.33)	2.55 (0.69)	0.4814
CSF IgA (mg/L)	4.16 (2.34)	4.80 (3.89)	4.71 (3.65)	4.25 (3.04)	2.88 (1.92)	0.5453
Q-IgA × 10^3^	1.79 (0.91)	2.21 (1.21)	1.78 (0.92)	1.97 (2.86)	1.37 (0.61)	0.8797
CSF IgM (mg/L)	0.32 (0.31)	0.62 (0.89)	0.40 (0.47)	0.35 (0.40)	0.29 (0.22)	0.2194
Q-IgM × 10^3^	0.31 (0.21)	0.53 (0.42)	0.35 (0.28)	0.33 (0.22)	0.31 (0.14)	0.0809
Leukocytes/μl	0.73 (1.10)	1.11 (0.78)	1.37 (1.40)	0.84 (1.27)	1.00 (1.20)	0.0912
Lactate (mmol/L)	1.79 (0.28)	1.74 (0.14)	1.87 (0.35)	1.78 (0.26)	1.75 (0.24)	0.4034

No difference in CSF parameters was observed across OCB type subgroups. Comparison by one-way ANOVA.

**Table 5:** Prevalence of infectious, inflammatory, metabolic and cardiovascular comorbidities in the amyotrophic lateral sclerosis (ALS) patients subgrouped according to the oligoclonal bands (OCB) pattern.

ALS patients (*n* = 457)						
	OCB type 1 (*n* = 130)	OCB type 2 (*n* = 9)	OCB type 3 (*n* = 35)	OCB type 4 (*n* = 274)	OCB type 5 (*n* = 8)	*p*-Value
Infections % (*n*)	0.77% (1)	11.11% (1)	8.57% (3)	0.36% (1)	0% (0)	*0.0001
Borrelia % (*n*)	0% (0)	11.11% (1)	0% (0)	0% (0)	0% (0)	*4.022e-10
Meningitis % (*n*)	0% (0)	0% (0)	5.71% (2)	0% (0)	0% (0)	*7.408e-05
Zoster % (*n*)	0.77% (1)	0% (0)	3.96% (1)	0.36% (1)	0% (0)	*0.5433
Inflammation % (*n*)	5.38% (7)	11.11% (1)	14.29% (5)	10.94% (30)	0% (0)	*0.2812
Multiple sclerosis % (*n*)	0% (0)	0% (0)	3.96% (1)	0% (0)	0% (0)	*<2.2e-16
COPD % (*n*)	0% (0)	0% (0)	5.71% (2)	2.19% (6)	12.0% (1)	*0.0408
Asthma % (*n*)	3.08% (4)	11.11% (1)	0% (0)	2.19% (6)	0% (0)	*0.3723
Arterial hypertension % (*n*)	29.23% (38)	33.33% (3)	48.57% (17)	31.75% (87)	50% (4)	*0.2051
Heart disease % (*n*)	8.46% (11)	11.11% (1)	11.43% (4)	12.41% (34)	0% (0)	*0.6603
Coronary heart disease % (*n*)	3.85% (5)	11.11% (1)	5.71% (2)	6.20% (17)	0% (0)	*0.7422
Stroke % (*n*)	0.77% (1)	0% (0)	3.96% (1)	0% (0)	0% (0)	*0.1781
Diabetes mellitus % (*n*)	13.08% (17)	11.11% (1)	11.43% (4)	8.39% (23)	0% (0)	*0.5312

A significant over-representation of infections and inflammation in patients with intrathecal synthesis (OCB 2 and 3) is detected. *Pearson’s *χ*^2^ test.

## Results

### Prevalence of oligoclonal bands and their subtype patterns is comparable in ALS and AD, MCI, TTH or FP

We set out to establish if any pattern of oligoclonal bands in CSF was enriched in ALS compared to four cohorts of patients diagnosed with AD, MCI, TTH or FP. The clinico-demographic and CSF features of the five groups are reported in Table 1. Overall, ALS, AD and MCI patients were comparable in age, whereas TH and FP were significantly younger; furthermore, ALS patients were more frequently male, whereas for AD, MCI, TH and FP the opposite was true. In respect to the CSF parameters, the five groups were significantly different for total CSF Albumin and the ratio of CSF/Serum Albumin (Q-Alb; *p* = 0.004 and *p* = 1.041e-05, respectively); *post hoc* comparisons revealed that this effect was largely due to the difference between TTH and AD (*p* = 8e-06). The five groups also showed a significant difference in Q-IgG and total CSF IgA (*p* = 0.0006703 and *p* = 0.004655). The *post hoc* analysis revealed that these differences were due to the reduced Q-IgG in the TTH patients compared to AD patients (*p* = 0.002) and in the slight decrease in CSF IgA in ALS patients compared to AD patients (*p* = 0.043); all other groups were pairwise comparable. Finally, a statistically significant difference was observed for lactate (*p* = 0.007), but the *post hoc* analysis revealed that this was solely due to the difference between AD and MCI (*p* = 0.026) whereas ALS patients were not different from all others.

The five patterns of OCB were unevenly represented (Table 2): in the ALS patients, the 60% displayed a type 4 pattern (similar OCB in serum and CSF, indicating systemic immune response), whereas only 28% displayed no OCB; a minority of patients displayed a type 3 pattern (OCB in serum in addition to those found in the CSF) and a very small subgroup, approx. 2%, displayed a type 2 pattern (OCB only in CSF, indicative of intrathecal immune response). Likewise, 2% of ALS patients displayed a pattern compatible with monoclonal gammopathy. Most importantly, the relative representation of the five OCB patterns was identical in the five cohorts (*p* = 0.22) and with no instance of higher-than-expected pattern representation. Thus, these data conclusively demonstrate that no pattern of OCB is enriched in ALS.

### Oligoclonal band subtypes do not affect onset and progression rate of ALS

Next, we investigated if subgroups of ALS patients with distinct OCB patterns also displayed specific clinical features (Table 3). A strong trend (*p* = 0.0077) toward a difference in the age of onset was largely driven by the small subgroup with monoclonal gammopathy (type 5), which were older than the others (in agreement with the higher incidence of gammopathies in older patients). A statistically significant difference (*p* = 0.037) was detected in the ALS-FRS at the first evaluation (sampling); *post hoc* analysis revealed that this difference was due to the significantly lower ALS-FRS score in patients with type 3 OCB vs. patients with type 1 (*p* = 0.031) and, at strong trend level, vs. patients with type 4 (*p* = 0.061). On the other hand, the five subgroups of ALS patients were comparable in terms of their site of onset and progression rate. The five groups were also largely comparable in terms of their CSF features (Table 4); strong non-significant trends were found for Q-IgM (*p* = 0.08, higher in the subtype 2) and for leukocytes (*p* = 0.09, higher in subtypes 2 and 3). Both these findings are consistent with the OCB patterns (type 2 and 3, OCB in CSF only or more OCB in CSF than in serum) indicating an immune activation affecting the intrathecal compartment. Thus, the presence and pattern of OCB is not associated with a distinct clinico-biological phenotype of ALS patients with the exception of a more severe ALF-FRS at presentation for patients with systemic and intrathecal immune activation, corresponding to OCB type 3.

### OCB subtypes do not predict overall survival

We tested if the OCB pattern was associated with a different survival. We considered either the overall cohort, using the last visit as a censoring event (Figure 1), or a subgroup of patients (222 subjects) for which death certificates are available (Figure 2). In both analysis (Kaplan–Meier Log-rank method), no statistical difference was observed across the five subgroups (overall cohort *p* = 0.46; cohort with death certificates *p* = 0.83); a trend toward shorter survival of patients with the monoclonal gammopathy type was observed, in agreement with the older age and the co-existence of a neoplastic condition. We also performed a Multivariate Cox Regression analysis to confirm the lack of effect of OCB subtypes: none of the considered potential confounders, sex (*p* = 0.40), BMI (*p* = 0.90), age of onset (*p* = 0.99), onset site (*p* = 0.3) was significantly modifying the lack of correlation between survival and OCB subtype.

### Isolated CSF OCB may result from unrelated comorbidities

Finally, we performed a survey of the possible sources of OCBs in ALS patients. We analyzed the prevalence of comorbidities possibly related to OCBs (infectious or inflammatory diseases) and comorbidities unrelated to OCBs (metabolic and cardiovascular conditions; Table 5). Infectious (*Borrelia Burgdoferi*, viral meningitis, herpes zoster being the most frequent) comorbidities were significantly over-represented in patients with OCB type 2 and 3 (*p* < 0.001); inflammatory conditions (systemic autoimmune diseases including thyroiditis, arthritis and, in one case, multiple sclerosis) were more prevalent in patients with OCB type 2 and 3 (11 and 14%) compared to patients without OCB, but the finding did not reach statistical significance (*p* = 0.23). Furthermore, Chronic Obstructive Pulmonary Disease (COPD) was significantly more prevalent in patients with OCB type 3 than in other subtypes (5.71%, *p* = 0.040). On the other hand, cardiovascular comorbidities, diabetes mellitus and asthma bronchiale were similarly distributed across the OCB subtype groups. Thus, OCB may originate as consequence of inflammatory or infectious comorbidities.

## Discussion

In the present work, we provide compelling evidence, based on a large prospective cohort of ALS patients, that OCB do not appear in increased frequency in ALS patients compared to other neurological controls and that OCB pattern, either related to a systemic or to a CNS immune activation, does not correlate with a distinct clinical phenotype in terms of onset, progression and overall survival. Thus, OCBs may appear in ALS as an unrelated phenomenon, irrelevant to the pathogenic process. This negative finding has implications for the clinical testing of immunomodulatory treatments for ALS and for the diagnostic interpretation of a positive OCB report.

Initial reports (26, 27) suggested that a subset of ALS patients may display OCB as part of their pathophysiology; however, these patients were also reported to have substantial increase in CSF albumin content and/or paraproteinemia (27). Thus, it remains possible that an alternative diagnosis, other than ALS, could be better suited for these cases; furthermore, some of the ALS cases with OCB displayed lymphoproliferative disorders (42). More recent work reported OCB prevalence in 9/259 [3.5%; (28)] and in 0.5–2% (40, 43). Our survey reveals a 9/457 (2%) prevalence for type 2 OCB, in line with previous reports, and a substantially higher prevalence (44/457, approx. 10%) when type 2 and type 3 are considered together. Since the prevalence appears comparable across the five conditions taken into consideration (ALS vs. AD, MCI, TTH and FP) as previously observed (40), the upward trend may be accounted for by changes in sensitivity of OCB detection and definition of positive OCB.

Our data show that ALS patients do not display higher incidence of OCB, in any pattern, than other neurological controls, suggesting that any inflammatory/autoimmune condition underlying the OCB may be coincidental rather than causal. The occurrence of inflammatory/autoimmune conditions in ALS patients has been previously subject to investigation in the Swedish nation-wide cohort; only 28 patients, out of 263 patients with purported co-existence of both conditions, were positively identified as affected by multiple sclerosis, myasthenia gravis, inflammatory polyneuropathies or dermatopolymyositis. The low rate of co-occurrence and the absence of effect on survival supported the hypothesis that the inflammatory condition did not substantially affect the progression of ALS (44) and may be due to chance or misdiagnosis (45, 46). Sporadic cases of ALS occurring together with neuromyelitis optica (47), autoimmune encephalitis (48) or multiple sclerosis (49) have been reported. Interestingly, OCB appears to be relatively more prevalent in ALS cases with distinct genetic causes [3/12 in genetic ALS cases vs. 6/247 in sporadic; the three cases presenting TDP-43 or ANG-2 mutations; (28)] and with distinctive presentation and clinical course (cerebellar involvement and hyperintense lesion in white matter). It must be noted that in these cases, the possibility of a misdiagnosis should be considered and solitary sclerosis has been identified in cases with clinical presentation compatible with motoneuron disease but positive OCB (45).

In those cases in which ALS co-occurred with an autoimmune disease and positive OCB, the clinical course of ALS was not substantially different from those of isolated ALS, suggesting that autoimmunity and OCB may originate from the release of antigens in the course of the neurodegenerative process or may occur by chance alone.

Moreover, patients with signs of intrathecal synthesis (OCB type 2 and 3) showed significantly more infectious and inflammatory comorbidities (including 1 case of a co-occurring diagnosis of Multiple Sclerosis). These findings further suggest that the detection of OCB may indicate an ongoing infectious or autoimmune disorder coincident, but unrelated to, ALS. Nevertheless, the coincident conditions determining the OCB may affect the overall performance of the patients: in fact, ALS patients with OCB type 3 display a lower ALS-FRS at CSF sampling (a proxy of ALS-FRS at diagnosis) than patients with no OCB (type 1) or intrathecal synthesis-only (type 2) or systemic-only activation (type 4; strong trend, *p* = 0.06), despite no difference in progression rate. It is possible that a co-occurring inflammatory systemic condition affecting the brain may reduce the overall fitness of patients and lower the ALS-FRS at diagnosis; this hypothesis may be tested in dedicated studies.

Furthermore, our data show that the presence or absence of OCB, or any pattern of OCB, does not affect progression rate and survival. Thus, our data suggest that the pathophysiological process highlighted by OCB may not be relevant to the pathogenic cascade of ALS. Our findings are consistent with the inefficacy of B-cell deletion to modulate the disease burden and survival of mutant SOD1 ALS mice (50), despite the appearance of autoantibodies in this model. Thus, the observed enrichment of B-cells in meningeal tissue of the mutant SOD1 ALS mouse model (12) may constitute a response to the neurodegenerative process but not a direct player in the loss of motoneurons. It is not possible to discount, though, that the immune response and B-cell activation may be more relevant in subsets of ALS patients characterized by specific genotypes: recently, clonally expanded CD8+ T cells have been identified in familial ALS-4 cases (4), and autoimmune response characterize a murine model lacking C9Orf72 protein expression (51); furthermore, OCB appears to be disproportionately more common in ALS patients with TDP-43 mutations (28). Also, sub-grouping of ALS patients may require the consideration of markers of signaling within specific subpopulations, such as IL-23 (29) or IL-6 (52).

Finally, our data do not completely discount the involvement, in the ALS pathogenesis, of other immune processes not recaptured by OCB detection or pattern. In fact, several adaptive and innate immunity abnormalities have been reported at the level of regulatory T cells and GATA3+ Th2 lymphocytes [reduced in patients with rapid progression (53–55)] and deregulated monocytes may infiltrate the spinal cord of ALS patients and murine models (56, 57). Furthermore, local immune responses involving reactive microglia may be involved in ALS pathophysiology (2, 6, 8) but still may not be highlighted by OCB.

In conclusion, the lack of correlation between OCB and progression or survival of ALS patients, and their likely origin as epiphenomena or coincidental conditions, suggests that clinical trials with anti B-cell immunomodulatory agents should be performed with caution and may have a lower priority compared to agents addressing other cellular branches of the innate and adaptive immune system. On the other hand, the detection of OCB in the CSF of ALS patients may be considered a warning sign of a co-occurring inflammatory or infectious disease and prompt the opportune evaluation; because of the comparatively small prevalence of comorbidities in ALS patients, this investigation is to be considered exploratory and may require re-assessment in a large cohort.

## Data availability statement

The raw data supporting the conclusions of this article will be made available by the authors, without undue reservation.

## Ethics statement

The studies involving human participants were reviewed and approved by Ethics committee of the Ulm University, No. 20/10. The patients/participants provided their written informed consent to participate in this study.

## Author contributions

VK organized the database, performed the statistical analysis, and contributed to the manuscript. SJ provided the clinico-demographic information about the AD and the MCI cohorts. JL provided the clinico-demographic information about the TTH and the FP cohorts. JD and JK contributed to the clinico-demographic information about the ALS cohort. HT contributed to the CSF datasets. VK, AL, and FR contributed to the conception and design of the study. AL, FR, VK, and HT contributed to the initial draft of the manuscript. VK, SJ, JL, JD, JK, HT, AL, and FR contributed to the final version of the manuscript. All authors contributed to the article and approved the submitted version.

## Funding

This work was supported by the German Center for Neurodegenerative Diseases (DZNE).

## Conflict of interest

The authors declare that the research was conducted in the absence of any commercial or financial relationships that could be construed as a potential conflict of interest.

## Publisher’s note

All claims expressed in this article are solely those of the authors and do not necessarily represent those of their affiliated organizations, or those of the publisher, the editors and the reviewers. Any product that may be evaluated in this article, or claim that may be made by its manufacturer, is not guaranteed or endorsed by the publisher.
