# Supplementary figures and images for: CSF oligoclonal IgG bands are not associated with ALS progression and prognosis
Source: Front Neurol. 2023 May 5;14:1170360. doi: 10.3389/fneur.2023.1170360 (PMC10196068; doi:10.3389/fneur.2023.1170360)

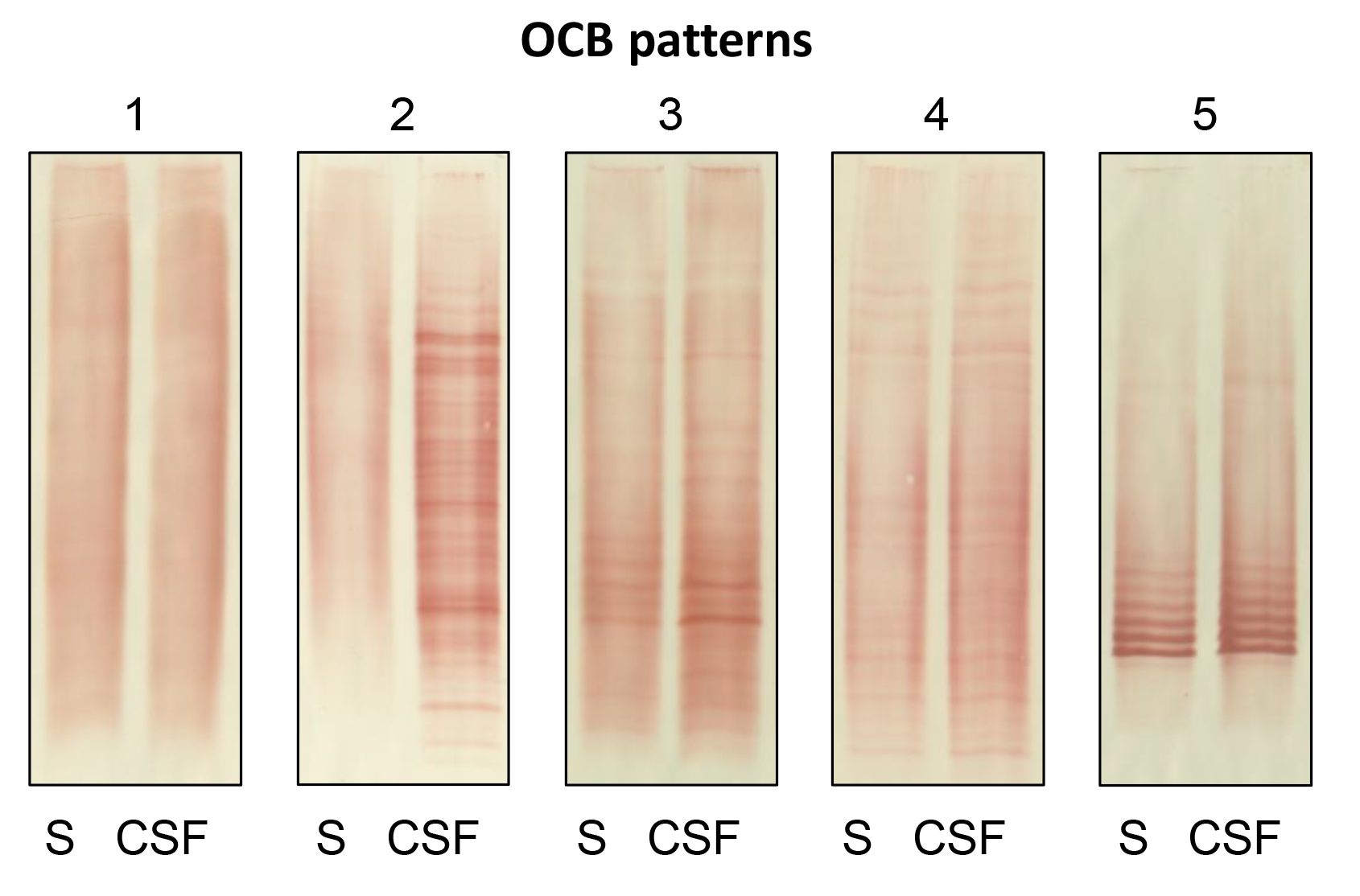

Supplement: Supplementary file 1 [file Image_1.TIF]
